# Expert consensus on clinical research nurse management in China

**DOI:** 10.1016/j.apjon.2023.100254

**Published:** 2023-06-05

**Authors:** Jieying Ge, Yanfei Liu, Bin Gan, Yuhong Liu, Xiaohong Liu, Jie Qiao, Qing Zhao, Ting Chang, Jing Wang, Juying Xing

**Affiliations:** a State Key Laboratory of Oncology in South China, Sun Yat-sen University Cancer Center, Guangzhou, China; b Fudan University Shanghai Cancer Center, Shanghai, China; c Guangdong Provincial People's Hospital, Guangzhou, China; d Zhongshan Ophthamic Center, Sun Yat-sen University, Guangzhou, China; e Peking University Cancer Hospital & Institute, China; f Jiangsu Cancer Hospital, Nanjing, China; g SMO ClinPlus Co., Ltd, China; h Shanghai Pulmonary Hospital, Shanghai, China; i Jilin Cancer Hospital, China

**Keywords:** Clinical research nurse, Clinical trials, Qualification, Management consensus

## Abstract

**Objective:**

With the rising number of clinical trials conducted in China, the role of Clinical Research Nurses (CRNs) within clinical trial organizations has become increasingly crucial. However, in the absence of industry guidelines, the management of CRNs lacks clarity. This study aims to address this gap by establishing a consensus on CRN management.

**Methods:**

The IACRN-Shanghai Chapter assembled a panel of experts to develop a consensus on the management of clinical research nurses. This consensus was formulated through an extensive literature review, expert evaluations, and collaborative conference discussions.

**Results:**

The consensus document offers a comprehensive professional definition of CRNs and provides detailed insights into their management. It covers aspects such as job settings, qualifications, responsibilities, training, assessment, workload management, staffing allocation, performance evaluation, and career progression.

**Conclusions:**

Establishing a professional definition for CRNs creates a standardized reference point for clinical trial institutions to effectively manage these professionals. Consistency in CRNs’ roles, training, staffing, and corresponding assessments is essential for promoting their sustainable and healthy development within the field of clinical research.

## Introduction

Clinical research in China has flourished over the past few years, and an increasing number of medical institutions have begun to stress quality and efficiency when conducting clinical research. It is imperative to organize a well-trained research team to ensure the smooth progress of clinical research in medical institutions and high project quality. In general, research teams are comprised of research doctors, pharmacists, clinical research nurses (CRNs), and clinical research coordinators (CRCs). CRNs are gradually becoming essential core participants in clinical research and are responsible for the coordination and management of clinical research projects and to carry out subject treatment according to the research plan.^1^ Because a record-keeping system has been implemented in clinical trial institutions,^2^ more and more medical institutions will begin to train full- or part-time CRNs. The initial group of CRNs in China joined the International Association of Clinical Research Nurses (IACRN) and established the China (Shanghai) branch. The IACRN-Shanghai Chapter regularly holds academic exchanges and training activities to discuss the progress of clinical research and nursing work with international peer experts. To promote the healthy development of clinical research nursing in China, the IACRN-Shanghai Chapter assembled expert teams to discuss the management problems of CRNs with respect to role definition, personnel employment, responsibilities, training, and performance appraisal. In addition, the expert teams reached a consensus to provide reference for domestic clinical trial institutions.

## Methods

### Drafting the consensus

#### Establishing the writing group

The consensus writing group consists of nine members, all of whom are senior CRNs with clinical research management experience in China, including two deputy chief nurses, four supervisors, one nurse, and one clinical research manager. The team members are from Beijing, Shanghai, Guangzhou, Nanjing, Changchun, and other cities. The members of the writing team are responsible for consulting, analyzing relevant documents, combining the practical experience of the team members, selecting the theme, and writing content to form a consensus draft.

#### Establish a review expert group

The review group consists of five experts with > 10 years of experience in clinical trials, clinical trial nursing, and clinical trial management. The representatives include two chief nurses, two associate researchers, and two chief nurses, four of whom are experts from the Chinese mainland. Another representative familiar with the field of international CRNs was invited from Taiwan, China.

### Form a consensus system of CRN management

#### Literature review

Literature data relevant to domestic and international CRNs were collected. The Chinese databases included CNKI, VIP, and Wanfang. The search terms included “research nurse” and “research nurse coordinator.” A total of 173 articles were searched; seven articles not related to the topic were excluded. The foreign language databases consulted included PubMed and Medline. The search terms included “clinical research nursing,” “clinical research coordinators,” “research nurse,” “clinical research nurse,” “clinical trial nurse,” “study coordinator,” and “research nurse coordinator” were the search terms; a total of 861 international articles were searched.

### Document arrangement and induction

International scholars have abundant research materials on clinical research nursing and CRNs. The literature mainly focuses on defining the scope of clinical research nursing practice^3^^,^^4^^,^^11^, ^12^, ^13^, ^14^ and evaluation of ability to guide the management of CRNs in China.^5^, ^6^, ^7^, ^8^, ^9^, ^10^^,^^15^ The literature published by Chinese scholars, however, has mainly focused on position recognition, job content, definition of CRN responsibilities, training, and the generation and development of CRN roles,^16^, ^17^, ^18^, ^19^ but there is a lack of research in CRN work quantification, manpower allocation, performance appraisal, and promotion.^18^ Most of the literature focuses on a specific topic, the correlation between the topics lacks research and elaboration, and the guidance for CRN management practice is limited. After transcribing the group discussion, it was decided that the consensus should focus on the overall elaboration of the key issues of CRN management and the significance of practical guidance.

### Draft of consensus

According to the retrieved literature, the writing team summarized, sorted, analyzed, screened, and refined the six key themes of CRN management, as follows: “role definition;” “position setting and qualification;” “responsibilities and tasks;” “training and assessment;” “job quantification and manpower allocation;” and “performance assessment and promotion.” The members of the writing team are responsible for writing one of the topics first, then three members summarize the topic. The writing group has conducted two rounds of group discussions on the first draft of the consensus. The first draft of the consensus is mainly about discussion, and the argumentative expression requires readers to take time to understand. After sorting out the internal relevance among the six themes, the writing team made a structured list of the content of each part in the second draft to more clearly demonstrate the intrinsic relevance of each topic in postmanagement. In the second round of discussion, the writing group will discuss the specific content of each topic one-by-one, and the corresponding items shall be reserved and adopted for expression, which shall be agreed upon by the writing group, and finally form a consensus draft.

### Expert discussion

After the first draft of the consensus is formed, it will be sent to five experts by mail for review. Five experts put forward opinions on each theme, including four opinions on the first-level theme and 25 opinions on each content under the first-level theme. According to expert opinions, the order and mode of first-level theme expression are adjusted, the second-level theme expression is streamlined, and the consensus content is adjusted.

### Soliciting opinions

Two rounds of meetings and discussions will be held within the IACRN Association. The annual meeting will be held in the form of an online meeting of the IACRN-Shanghai Chapter on October 20, 2020. The participants include 70 members of the IACRN-Shanghai Chapters. The participants discussed the official content of the consensus together and collected four opinions for modification. At the annual meeting held in Nanjing, China, on October 30, 2021, the clinical nursing managers of the annual meeting clarified the corresponding concepts and answered the participants' questions.

## Definition of a CRN

### CRN definition

A CRN refers to a member of the nursing staff with a nursing professional education background and nursing practice qualifications who participates in clinical research-related work (ie, engaged in clinical research project management and coordinating research parties, and performing subject treatment in accordance with the research protocol). CRNs work primarily in clinical trial institutions, placing a focus on the balance between subject protection and research protocol adherence, and solving issues involving clinical care feasibility and clinical research compliance.^1^

## Postsetting and qualification of CRNs

### Postsetting of a CRN

Medical institutions conducting clinical research are suggested to subdivide the nursing team and set up full- or part-time CRNs, with reference to the training of specialist nurses.^17^ According to the hospital or department management mode, the number and complexity of clinical research, the population demographics of research subjects and other aspects, and the number of personnel who need to set up CRNs is determined.^20^

### Postsetting method

#### Part-time

Departments or hospitals with a small number of clinical studies require part-time CRNs who are usually clinical nurses.^17^

#### Full-time

Departments or hospitals with complex or considerable clinical research projects require full-time CRNs who are external recruitment/separate professional CRNs from existing caregivers and can be classified in one of three management modes as follows.

1) managed by professional groups or departments;
2) managed by the institution office/clinical research center; and
3) managed by the nursing department.

### Basic qualifications

#### Education background and professional requirements

A college degree or above is required with a major in clinical nursing, a nurse qualification certificate, and registration in medical institutions.

#### Language requirements

An ability to read and write in Chinese and English to meet the needs of clinical research is required.

#### Office software capability

The necessary skills in using office software and office equipment, and proficiency in using EXCEL, WORD, and other office software are required.

## Responsibilities and tasks of CRNs

### Responsibilities and task framework of CRNs

Clarifying the job responsibilities and tasks of CRNs established the basis for the training, manpower allocation, and performance evaluation. One CRN job responsibility category has 15 responsibilities under several tasks.^17^^,^^22^ The second category involves the clinical research development stage^21^ and has five stages, including basic work, before start-up, start-up stage, project implementation, and close-out. At each stage, the CRN should undertake different responsibilities and tasks. Based on the CRN responsibilities and task framework table, all the categories of CRN responsibilities are listed, and the work tasks of research nurses in different research processes are specified (Table 1).

**Table 1:** Responsibilities and tasks of clinical research nurses.

Order number	Classification of responsibilities	Work tasks	Basic work	Before start-up	Start-up phase	Project implementation	Close-out stage
						Screening/enrollment/visit/follow-up	
1	Facilities management	• Confirm the routine equipment maintenance and calibration work of the institution	√				
		• Obtain the annual quality inspection report of common equipment in time	√				
		• Confirm whether the equipment required for project implementation is complete		√			
		• Confirm with PI and the sponsor whether additional equipment is required according to the project requirements		√			
		• Manage the project specific equipment provided by the sponsor according to the hospital regulations			√	√	
		• Return the special equipment provided by the sponsor					√
2	Training and education	• Complete training on laws and regulations related to clinical research	√				
		• Understand clinical research methods and basic knowledge of Statistics	√				
		• Gain insights into the basic knowledge of disease medicine related to clinical research	√	√	√	√	
		• Have communication, foreign language, and computer skills	√	√	√	√	√
		• Improve the necessary nursing professional knowledge and skills for clinical research	√			√	
		• Project training (eg, program and SOP)		√			
3	Project planning and implementation	• Engage in a wide variety of research meetings, master clinical research requirements, and follow-up research progress		√			
		• Assist PI in formulating/reviewing research protocol, informed consent, CRF, and other documents	√	√			
		• Project initiation application and follow-up		√			
		• Ethics committee review application and follow-up		√			
		• Human genetic resources application and follow-up		√	√	√	
		• Contract review application and follow-up		√			
		• Assist investigator to organize research teams according to project needs		√			
		• Work coordination and application of relevant departments in the research center			√		
		(eg, pathology examination imaging and pharmacy)					
		• Assist in preparing, organizing, and holding kick-off meetings			√		
		• Confirm that the members of the research team have been trained and authorized		√	√	√	
		• Contrive and continuously optimize the project implementation process		√	√	√	
		• Develop and continuously optimize worksheets			√	√	
		• Assist the research team in discussing drug combinations and treatment plans				√	
		• Record and report the scheme violations and continuously optimize the implementation process				√	
		• Submission of annual reports^24^				√	
		• Submission of protocol and revision of informed consent			√	√	
		• Review/submit CRF, drug test report, IB, and other project-related documents				√	√
4	Subject management	• Assist in the recruitment of subjects				√	
		• Assist in screening subjects				√	
		• Informed consent in the scope of professional qualification				√	
		• Check the eligibility of subjects				√	
		• Complete the randomization/enrollment procedure of subjects				√	
		• Appointment and arrangement of visit procedures (eg, examination, treatment, and follow-up of subjects)				√	
		• Offsite communication and follow-up via telephone or other means of communication				√	
		• Tracking and recording of combined treatment and medication of subjects in the study period				√	
		• Carefully observe the tracking and recording of the subjects' condition changes				√	
		• Survival follow-up				√	
5	Subject education	• Instruct subjects to comply with the protocol for follow-up				√	
		• Instruct the subjects to use the trial drug				√	
		• Educate the subjects on prohibited drugs and treatment				√	
		• Instruct the subjects about diet, exercise, and other disease-related knowledge				√	
		• Instruct the subjects to use the relevant equipment for the test				√	
		• Instruct subjects to complete diaries and questionnaires				√	
6	Safety monitoring	• Finding and observing adverse events of subjects				√	
		• Assist in collecting, reporting, tracking, and recording security incidents				√	
		• Assist researchers in coping with adverse events				√	
		• Provide adverse event nursing guidance and education to subjects				√	
		• Timely report SAE				√	
		• Timely submission of SUSAR to ethic committee				√	
7	Investigational drug management	• Assist in the management of the central pharmacy/satellite pharmacy of the experimental institution		√			
		• Ensure that the storage environment of the test drug meets the requirements			√		
		• Receipt, storage, distribution, and return/destruction of investigational drugs			√	√	
		• Receiving, configuration, and use of investigational drugs				√	
		• Storage of investigational drugs in clinical departments, warehousing registration, and return				√	
		• Drug dose calculation and verification				√	
		• Instruct subjects to fill in medication records			√	√	√
		• Medication compliance management				√	
		• Train the executors of the investigational drug (dispensing and injection) according to the program			√	√	
		• Collect drug test reports			√	√	
		• Timely replenish the inventory of investigational drugs in the validity period			√	√	
8	Investigational device/reagent management	• Ensure that the storage environment of investigational devices/reagents meets the requirements				√	
		• Receive, manage, and return investigational devices/reagents according to hospital management requirements			√	√	
		• Requisition and use of investigational devices				√	
		• Receive training on the use of investigational devices/reagents			√	√	
		• Inventory management, stock replenishment application, and warehouse in and warehouse out registration				√	
		• Counting, destruction, recycling, and recording of test reagents					√
9	Sample management	• Develop sample management process		√			
		• Receiving, checking, using, and warehousing management of sample-related consumables			√		
		• Sample collection/collection, processing, storage, transfer, and warehousing registration				√	
		• Records of sample management process				√	
10	Data management	• Discuss and confirm the source data File of the research project with the researchers			√		
		• Issue, recover, and save diary cards and questionnaires				√	
		• Fill out the CRF				√	
		• Answer data queries				√	√
		• Research-related data copy and transmission				√	
		• Complete first level quality control					
		• Ensure data authenticity, logic, and other quality management work				√	
11	Contracts and funds	• Project budget		√			
		• Contract review, submission, and signing		√			
		• Fund injection and expenditure management			√	√	
		• Reimbursement and management of subjects' related expenses				√	
		• Collect and save bills related to project funds				√	
		• Accounting of project funds					√
12	Essential documents	• Confirm essential document management requirements	√	√			
		• Investigator folder preparation			√		
		• Collect, save, maintain, and update research process documents, and original data documents				√	
		• Filing and return of research documents					√
13	Material handling	• Management of project-related materials (eg, document supplies and consumables)			√	√	√
		• Confirm and supplement corresponding materials with the sponsor in accordance with the research requirements		√			
		• Manage materials according to hospital regulations, report registration, as well as use records			√	√	
		• Regularly check inventory and effectiveness and timely replenish			√	√	√
		• Timely liquidation and return of relevant materials					√
14	Communication and coordination	• Coordinate and communicate with institutional offices and ethics committees		√	√	√	√
		• Coordinate and communicate with relevant departments within institution (pharmacy, finance, information, inspection, and other medical technology departments)		√	√	√	√
		• Coordination and communication with the research team		√	√	√	√
		• Be responsible for coordinating and communicating with the sponsor and contract research organization		√	√	√	√
		• Coordination and communication of supervision, inspection, internal inspection, and verification			√	√	
		• Find and deal with operation problems in time			√	√	
		• Evaluate the operation process and improvement of the test institution (system construction)	√	√	√	√	
15	Research-related caregiving	• Research-related nursing physical examination, treatment, medication, and care				√	
		• Condition observation and psychological care				√	
		• Health education for patients and their families related to diseases and research				√	
		• First aid care				√	
		• Research ward nursing					

PI, principal investigator; SOP, Standard Operation Procedure; CRF, case report form; IB, Investigator’s Brochure; SUSAR, suspected unexpected serious adverse reaction; SAE, severity adverse event.

### Setting principles of the responsibilities of CRNs

1) The work tasks undertaken by CRNs should be delegated by the principal investigator (PI).
2) The duties of CRNs shall not exceed the scope of nursing qualifications.

### Factors influencing the responsibility setting of CRNs

#### Clinical study type

In all types of clinical research, the work of CRNs should be subject-centered, with equal emphasis on nursing care and research implementation management. CRNs also have different focuses in different types of research. In phase I clinical research, the work of CRNs paid more attention to the medication compliance of subjects, sample collection and management, and observation and collection of adverse events.^8^^,^^23^ In phase Ⅱ/phase Ⅲ/phase IV trials, the data management workload of CRNs was larger.^9^

#### Management of clinical trial institutions/departments

The management mode selected by the respective institution/department and its development stage are different, and there is a significant difference in the job responsibilities and tasks undertaken by CRNs in different institutions. In general, CRNs managed by clinical wards/departments focus on the nursing work of clinical research (eg, subject sample collection and administration). Central CRNs managed by the institutional office/clinical research center place a focus on the entire process management of the project and coordinate with all departments in the hospital. Moreover, CRNs, under the centralized management of the nursing department, will account for the entire process management of the project and the nursing work (eg, drug administration and sample collection) during the subject study while paying attention to the development of the clinical nursing discipline.

## Training and examination of CRNs

### Knowledge and ability of CRNs

Five types of knowledge and abilities are required for CRNs, including medical nursing, statistical skills, subject protection, compliance with laws and regulations, research management expertise, project implementation practical skills, and professional quality.^17^ The content covered by the above five types of knowledge and abilities, and their corresponding learning resources are listed in the CRN knowledge and ability framework model of this consensus. For more details, please refer to Table 2.

**Table 2:** Framework model for knowledge and ability of clinical research nurses.

Category	Knowledge	Primary	Intermediate	Senior	Reference resources
Medical care and statistics	Basic knowledge of medicine and nursing	√			College courses Guidelines for diagnosis and treatment of various diseases
	Research design and methods		√		General Good Clinical Practice training and clinical research/biostatistics-related textbooks
	Clinical research data management		√	√	General Good Clinical Practice training and clinical research/biostatistics-related textbooks
	Basis of biostatistics		√	√	General Good Clinical Practice training and clinical research/biostatistics-related textbooks
Subject protection and regulatory compliance	Regulations on the administration of drug clinical trial institutions		√		General Good Clinical Practice training and NMPA website
	Code for quality management of clinical trials of medical devices	√			General Good Clinical Practice training and NMPA website
	Code for quality management of drug clinical trials	√			General Good Clinical Practice training and NMPA website
	Measures for ethical review of biomedical research involving human beings	√			General Good Clinical Practice training and NMPA website
	Measures for the administration of drug registration		√	√	General Good Clinical Practice training and NMPA website
	Regulations of the people's Republic of China on the administration of human genetic resources		√	√	General Good Clinical Practice training and NMPA website
	Management measures for conditions and filing of medical device clinical trial institutions		√	√	General Good Clinical Practice training and NMPA website
	Drug Administration Law		√	√	General Good Clinical Practice training and NMPA website
	Administrative measures for registration of *in vitro* diagnostic reagents	√			General Good Clinical Practice training and NMPA website
	Declaration of Helsinki	√			General Good Clinical Practice training and NMPA website
Research management expertise	Facilities management		√		Industry consensus
	Planning and implementation	√			Manual For Clinical Trials Nursing
	Subject management	√			Manual For Clinical Trials Nursing
	Subject education	√			Manual For Clinical Trials Nursing
	Safety monitoring	√			Manual For Clinical Trials Nursing
	Investigational drug management	√			Manual For Clinical Trials Nursing
	Management of investigational devices and reagents	√			Manual For Clinical Trials Nursing
	Sample collection and management	√			Manual For Clinical Trials Nursing
	data management	√			Manual For Clinical Trials Nursing
	Contract and fund management		√		Manual For Clinical Trials Nursing
	Essential documents	√			Manual For Clinical Trials Nursing
	Material management	√			Manual For Clinical Trials Nursing
	Communication and coordination	√	√		Manual For Clinical Trials Nursing
	research-related caregiving	√			Manual For Clinical Trials Nursing
Practical skills of project implementation	Start basic work	√			Manual For Clinical Trials Nursing
	Various review applications and follow-up of the project	√			Manual For Clinical Trials Nursing
	Project start-up work	√	√	√	Manual For Clinical Trials Nursing
	Subject recruitment practice	√			Manual For Clinical Trials Nursing
	Subject screening and informed consent	√			Manual For Clinical Trials Nursing
	Enrollment visit of subjects	√			Manual For Clinical Trials Nursing
	Subject visit management	√			Manual For Clinical Trials Nursing
	Close-out management	√			Manual For Clinical Trials Nursing
	CRF entry	√			Manual For Clinical Trials Nursing
	Nursing skills related to clinical research	√			Manual For Clinical Trials Nursing
Professional quality	Communication and coordination ability	√			Practical learning and teaching
	Planning, organization and management ability	√			Practical learning and teaching
	Educational ability		√		Practical learning and teaching
	Critical thinking		√		Practical learning and teaching
	Cautious independence spirit		√		Practical learning and teaching
	Continuous learning ability		√		Practical learning and teaching
	Teamwork spirit		√		Practical learning and teaching

NMPA, National Medical Products Administration; CRF, case report form.

#### Medical, nursing, and statistical skills

In addition to acquiring the basic knowledge of medicine and nursing, CRNs should master clinical research knowledge in the field of diagnosis and treatment of diseases and special nursing skills, and acquire the knowledge of clinical research design and methods in biological statistics, as well as the basic knowledge and concept of clinical research data management.

#### Subject protection and regulatory compliance

It is necessary to master the domestic laws, regulations, and international guidelines regarding subject protection and clinical research, including the International Council for Harmonization of Technical Requirements for Pharmaceuticals for Human Use (ICH) guidelines, Helsinki Declaration, “Quality Management Specification for drug clinical trials,” “ethical review measures for biomedical research involving human beings,” “regulations of the People's Republic of China on the administration of human genetic resources,” and “measures for the administration of drug registration."

#### Research management expertise

Research management expertise comprises equipment management, planning and implementation, subject management, subject education, safety management, investigational product management, sample collection, data management, contract and cost management, document management, material management, communication, and coordination, as well as project nursing.

#### Practical skills of project implementation

The practical skills of CRN project implementation are detailed as starting basic work, project review application and follow-up, project start-up, subject recruitment practice, subject screening and informed consent, subject enrollment visit, subject visit management, project conclusion management, CRF entry, as well as research-related nursing skills.

#### Professional quality of clinical research nurses

The following professional quality training is required for qualified new CRNs to ensure competency.^15^^,^^17^

##### Communication and coordination skills

CRNs should communicate and coordinate with researchers, clinical nurses, sponsors, central laboratories, subjects, CRC, and a wide range of departments in the hospital to ensure the implementation of clinical research.

##### Ability to plan, organize, and manage

CRNs participate in all aspects of clinical research, organize project preparation and start-up, subject screening, enrollment and follow-up, and supervision, inspection and onsite verification of superior competent departments during clinical research to ensure the orderly progress of the project.

##### Education ability

CRNs, facing subjects, clinical nurses, and CRC, are required to conduct education regarding clinical research (eg, the possible adverse reactions of experimental drugs, the drug administration process of clinical research, as well as the process of hospital and department work system).

##### Evaluation thinking

CRNs should evaluate the feasibility of the research plan based on their own clinical experience, legal and ethical principles, and medical theoretical knowledge and propose opinions to facilitate the smooth implementation of clinical research.

##### Spirit of cautious independence

CRNs should operate in strict accordance with the plan and different standard operating procedures when implementing the clinical research plan. CRNs should operate in accordance with the relevant procedures of the research report when there are significant differences between the plan and the clinic, and they cannot do it at will.

##### Continuous learning ability

With the development of new drugs in extensive fields and the continuous emergence of novel treatment schemes, CRNs are required to constantly learn and update their knowledge system to solve various problems in clinical research.

##### Team spirit

Clinical research requires the cooperation of CRNs and researchers, CRC, clinical nurses, inspectors, and many other members to ensure the smooth progress of clinical research with quality and quantity.

### Ways to CRN training

#### Training methods

Training methods include self-study, centralized teaching, practice with teaching, as well as selecting one or more ways based on different knowledge and skills to complete the corresponding content of training and learning.

#### Applicable objects

CRNs with different professional titles require different knowledge and abilities to facilitate hierarchical training and evaluation of CRN post-abilities (eg, primary, intermediate, and advanced). CRNs who master intermediate or advanced knowledge and skills can reserve personnel for the management of clinical trial institutions. Table 3 lists the job skill requirements of research nurses with different professional titles.

**Table 3:** Job skill requirements of clinical research nurses with different professional titles.

Professional ranks and titles	Knowledge and skills	Specific requirements
Primary	Facilities management Training and education Project planning and implementation Subject management Safety monitoring Investigational drug management Investigational device/reagent management Sample management Data management Contract and funding Management Essential document Material handling Communication and coordination Nursing skills required for the program	• Obtain the annual quality inspection report of the common test equipment in a timely fashion. • Confirm whether the equipment required for project implementation is complete • Check with the PI and the sponsor whether the supplementary equipment is required according to the project requirements • Return the special equipment provided by the sponsor • Complete training on laws and regulations related to research • Be familiar with the basic knowledge of disease medicine related to research • Have communication skills, foreign language skills and computer skills • Improve the necessary nursing professional knowledge and skills for clinical research • Engage in various research meetings, master the test requirements, and follow-up the research progress • Project initiation application and follow-up • Ethics committee review application and follow-up • Contract review application and follow-up • Application for internal platform resources in the Research Center • (eg, pathology, examination, imaging, and pharmacy)" • Assist in preparing, organizing, and holding the kick-off meeting • Confirm that the members of the research team have been trained and authorized • Contrive and continuously optimize the project implementation process • Develop various worksheets • Assist in recording and reporting protocol violations • Assist in submitting annual reports • Submit CRF, drug test report, IB, and other project-related documents • Assist in reviewing/submitting project summary and summary report • Assist in screening subjects • Informed consent in the qualification • Complete the randomization/enrollment procedure of subjects • Appointment and arrangement of visit procedures (eg, examination, treatment, and follow-up of subjects) • Offsite communication and follow-up by telephone or other means of communication • Tracking and recording of concomitant treatment and medication during the study of subjects • Closely observe the tracking and recording of the patient's condition changes • Survival follow-up • Subject education • Finding and observing adverse events of subjects • Assist in collecting, reporting, tracking, and recording security incidents • Assist researchers in timely reporting SAE • Assist researchers to submit SUSAR in time • Receipt, storage, distribution, return/destruction of drugs • Receiving, configuration and use of trial drugs • Storage of trial drugs in clinical departments, warehousing registration, and return • Drug dose calculation and verification • Instruct subjects to fill in medication records • Medication compliance management • Obtain drug test reports regularly • Check the expiry date of the trial drug and replenish the inventory in time • Ensure that the storage environment of test instruments/reagents meets the requirements • Receive, manage and return test instruments/reagents according to hospital management requirements • Receiving and use of test instruments • Receive training on the use of test instruments/reagents • Inventory management, stock replenishment application, and warehouse in and warehouse out registration • Assist in the counting, destruction, recycling, and recording of test reagents • Receipt, inspection, use and warehousing management of consumables related to samples • Sample collection/collection, processing, storage, transfer, and warehousing registration• records of sample management process • Issue, recover, and save diary cards and questionnaires • Fill out the CRF • Answer data queries • Research-related data copy and transmission • Contract review, submission, and signing • Fund injection and expenditure management • Reimbursement and management of subjects' related expenses • Collect and save bills related to project funds • Confirm project document management requirements • Investigator folder preparation • Collect, save, maintain, and update clinical research process documents and original data documents • Filing and return of research documents • Management of project-related materials (eg, document supplies and consumables) • Confirm and supplement corresponding materials with the sponsor in accordance with the requirements of clinical research • Regularly check inventory and effectiveness and timely replenishment • Timely liquidation and return of relevant materials • Coordinate and communicate with institutional offices and ethics committees • Coordination and communication with the research team • Coordinate and communicate with the sponsor and CRO/SMO • Coordination and communication of supervision, inspection, internal inspection, and verification • Identify and solve operation problems in time • Nursing physical examination, treatment, and medication nursing related to clinical research • Condition observation and psychological care • Health education for patients and their families related to diseases and research • Nursing work in experimental ward
Intermediate	Facilities management Training and education Project planning and Implementation Subject management Safety monitoring Investigational drug management Sample management Data management Contract and fund management Material management Communication and coordination Nursing skills	• Confirm the routine equipment maintenance and calibration work of the test institution • Manage the project specific equipment provided by the sponsor according to the hospital regulations • Understand clinical research methods and statistics • Project training (eg, program and SOP) • Assist PI in formulating/reviewing research protocol, informed consent, CRF and other documents • Human genetic resources management • Assist researchers to set up research teams according to project needs • Contrive and continuously optimize the project implementation process • Develop and continuously optimize research work forms • Organize the research team to discuss the combined medication and treatment plan • Follow up the plan violation and continuously optimize the implementation process • Write/review annual reports • Review the project summary and summary report • Assist in the recruitment of subjects • Check the qualification of subjects • Assist researchers in dealing with adverse events • Provide adverse event nursing guidance and education to subjects • Assist in the management of the central pharmacy/satellite pharmacy of the experimental institution • Ensure that the storage environment of the test drug meets the requirements • Train the executors of the trial drugs (dispensing and injection) according to the scheme • Develop sample management process • Discuss and confirm the source data file of the research project with the researchers • Ensure data authenticity, logic, and other quality management work • Accounting of project funds • Manage materials according to hospital regulations, report registration and use records • Internal platform department coordination and affairs communication of the test institution (affairs communication of pharmacy, finance, information, inspection, and other medical technology departments) • Emergency care
Senior	Project planning and implementation Contract and fund management	• Assist PI in formulating/reviewing research protocol, informed consent, CRF and other documents • Complete first level quality control • Project budget • Evaluate the operation process and improvement of the test institution (system construction)

PI, principal investigator; SOP, Standard Operation Procedure; CRF, case report form; IB, Investigator’s Brochure; SAE, severity adverse event; SUSAR, suspected unexpected serious adverse reaction; CRO, contract research organization; SMO, site management organization.

#### Training reference resources

Training resources are made available in the field of clinical research.

## Work quantification and manpower allocation of CRNs

### CRN job quantification

Quantifying CRN work can be calculated from three levels (macro, micro, and accurate calculation). Macroquantitative indicators include the number of trials and enrollments, which are easy and convenient to calculate. Microquantitative indicators include the number of screenings, enrollments, treatment visits, and follow-up visits, which can quantify the workload of CRNs more specifically. More accurate quantitative indicators take the number of tasks as the indicator to count the specific workload completed by CRNs during a specific period (eg, the number of informed consents, the amount of document management, the amount of sample collection and processing, the number of SAE reports, as well as the number of CRF filling). The precise calculation of the CRN workload is dependent on a mature management system or a powerful information management system. CRN workload calculation takes on a critical significance in reasonable manpower allocation.^18^^,^^20^

### Human resource allocation of CRNs

(1) To reasonably calculate the manpower allocation of CRNs, the job responsibilities should be reasonably divided according to the specific situation. Although Part 4 of this consensus lists the job responsibilities and tasks of CRNs in detail, various institutions can define the scope of work suitable for CRNs in each institution according to their own management mode. The scope of the work is different, and the CRNs are different. (2) Calculate the average working time of the respective task undertaken by CRNs in the project/visit. (3) Calculate the number of clinical research projects, enrollment, visits, and various work tasks undertaken by the employing unit. (4) The manpower allocation of CRNs required in the corresponding employing unit can be estimated according to the number of work tasks and the average working time.

### Expenditure of human resources allocation

The training and allocation of CRNs need human resources. CRNs are mainly responsible for completing clinical research projects, and the employment funds should mainly come from clinical research funds. First, the total amount of clinical research work of the employing unit should be reasonably calculated, and it is necessary to determine the manpower allocation that needs to be invested to clarify the personnel cost in the clinical research contract.

### Workload and performance incentive

The change in manpower configuration often cannot keep up with the dynamic change of clinical research workload. In practice, perfect planning of the CRN fixed workload cannot be done. It is imperative to motivate personnel to bear the load beyond the standard workload, as well as to set the workload and performance incentive mechanism.

## Performance evaluation and promotion of CRNs

### Performance evaluation indicators

Postevaluation of CRNs includes postcompetency and job performance evaluations. The evaluation of postcompetence involves basic medical knowledge and clinical research specialist knowledge, work skills, and professional quality, which is primarily completed during posttraining. Postperformance evaluation comprises the evaluation of the workload, work quality, and work progress of CRNs.

#### Workload

The workload of CRNs can evaluate the workload of different CRNs by comprehensively calculating the number of trials, number screenings, number enrollments, treatment visits, sample collection, number of processing visits, and SAE reports over a period.

#### Work quality

The work quality evaluation indicators of CRNs focus on the project quality, which can be determined by each institution according to the management direction. The indicators to evaluate the quality of the project include authorization error rate, number of missing trainings, number of protocol violation, medication compliance, and SAE report delay rate, as shown in Table 4.

**Table 4:** Requirements for job performance indicators.

	Workload	Working quality	Schedule
Evaluation indicators/content	• Number of trials • Number of enrollments • Number of screenings • Number of visits • Number of survival visits • Number of responsibilities • Number of single tasks	• Authorization error rate, number of missing trainings • Number of protocol deviations • Report submission delay • enrollments error • Visit over window rate • Adverse event record missing • Loss of survival follow-up • Medication compliance • Completion rate of diary card or questionnaire • SAE underreporting and delay rate • Missing records	• Project initiation progress • Ethics committee Review progress • Contract review progress • Data entry progress
How to evaluate	Workload data statistics	According to monitoring report inspection report quality control report	According to monitoring report
Who to evaluate	Managers of directly affiliated departments	PI, department managers	PI, department managers

SAE, severity adverse event; PI, principal investigator.

#### Work progress

It should also be an auxiliary target for the work evaluation of CRNs. The main evaluation indicators involve project approval progress, ethics committee review, follow-up progress, contract review progress, and data entry progress.

#### Evaluation method

The workload of CRNs is evaluated through manual statistics or a management system summary. The work quality can be evaluated by reporting to the ethics committee or by indicators (eg, the incidence of protocol deviations, SAE report delay rate, and data entry delay rate obtained during the quality inspection).

#### Evaluation personnel

In general, the personnel management department is responsible for the workload evaluation, and the work quality and progress are primarily evaluated by the PI or the directly affiliated management department.

### Promotion of CRNs

#### Title promotion

In general, clinical nursing title is divided into primary, intermediate, and senior, which are nurses, deputy director of nurses, and director of nurses, respectively. Each institution has different requirements for professional title evaluation. It is suggested that CRN title should be included in the professional title evaluation system of clinical nursing series, and the corresponding evaluation system should be established. Due to the employment mode, if it cannot be included in the nursing professional title evaluation system, the promotion channels can be set according to the primary, intermediate, and senior levels. The evaluation of CRNs with different professional titles/levels is listed in Table 5.

**Table 5:** Evaluation and promotion conditions of clinical research nurses with different professional titles.

The relationship between professional title and evaluation			
Content of examination	Primary	Intermediate	Senior
Nursing qualification examination	Pass the primary care health qualification examination	Pass the intermediate care health qualification examination	None
CRNs' Job skill examination	Pass primary job skill examination	Pass intermediate job skill examination	Pass advanced job skill examination
Workload evaluation	Number of trials Number of enrollments Number of visits	Number of items Number of groups Number of visits	Number of items Number of groups Number of visits
	To complete a specific amount of work in a time period, select quantitative indicators (eg, the number of trials, the number of visits, and the number of tasks). The management department is capable of determining the quantity requirements based on the situation of the respective test institution		
Other abilities evaluation	• English skill evaluation • Scientific research evaluation: the management department can determine whether the corresponding professional title requires a certain number of papers to be published in accordance with the specific situation of the respective experimental institution.		

#### Position promotion

As the institutions of full-time CRN team gradually grow, each clinical trial institutions can set the corresponding management positions according to the respective management to provide competent CRNs promotion space (eg, team leader and head nurse position) and set the corresponding evaluation requirements according to different positions.

## Discussion

CRNs have a developing role in China, which is different from the clinical research experience in European and American countries and regions. CRNs in European and American countries are employed by clinical research institutions or PIs. The scope and competency of CRNs has been explored and set CRNs as the professional discipline of nursing.^3^, ^4^, ^5^ In China, however, most clinical research institutions are public hospitals and the employment of personnel is limited by the quota. At first, research institutions or PIs employed CRNs to help manage clinical research. Due to the rapid growth in the amount of clinical research in the past 5 years, the quota cannot be provided. There are enough institutions to employ CRNs. A large number of externally employed CRC are used in clinical research, which makes the role of nurses in clinical research unclear.^25^^,^^26^

Chinese hospital managers have gradually found that the external employment CRC has relatively large mobility and began to reexplore the experience of using CRNs. Inexperienced hospital managers are generally confused about the definition of responsibilities of CRNs and ordinary clinical nurses, CRC, and how to cultivate them. Inexperienced hospital managers also pay more and more attention to the deep-seated problems of “survival” in positions, such as job assessment, human cost accounting, and performance evaluation.^18^ In different clinical research institutions, the actual management situation is very different, which makes CRNs, as new posts, face different difficulties in their development.

We hope to systematically summarize the existing explorations of clinical research nursing researchers and introduce the practical experience of pioneers by drawing up a consensus. In the consensus, there is internal correlation among various topics and most of the clinical research environment in China should be considered as far as possible. We do not think that CRNs must have the same responsibilities in all scenes. Hospital managers can choose the responsibilities of CRNs matching the management mode according to actual needs, and their training, issues such as workload assessment and manpower allocation should be set around the selection of responsibilities.

It is suggested that hospital managers and nursing managers should see that nurses can play an advantageous role in clinical research, develop more important roles of nurses in scientific research, and promote the development of nursing departments. As a framework blueprint, we will guide the follow-up work of the association and look forward to promoting the specialized development of clinical research and nursing.

## CRediT author statement

**Yanfei Liu:** Methodology, Writing – Review and Editing, Supervision.

**Jieying Ge:** Methodology, Formal analysis, Writing – Original Draft.

**Bin Gan:** Methodology, Writing – Review and Editing Original Draft.

**Yuhong Liu:** Methodology, Writing – Review and Editing Original Draft.

**Xiaohong Liu:** Methodology, Writing – Review and Editing Original Draft.

**Jie Qiao:** Methodology, Writing – Review and Editing Original Draft.

**Qing Zhao:** Methodology, Writing – Review and Editing Original Draft.

**Ting Chang:** Methodology, Writing – Review and Editing Original Draft.

**Jing Wang:** Methodology, Writing – Review and Editing Original Draft.

**Juying Xing:** Methodology, Writing – Review and Editing Original Draft.

All authors had full access to all the data in the study, and the corresponding author had final responsibility for the decision to submit for publication. The corresponding author attests that all listed authors who meet authorship criteria are included.
